# Leishmaniases: Strategies in treatment development

**DOI:** 10.1051/parasite/2025009

**Published:** 2025-03-05

**Authors:** Alissa Majoor, Grégory Michel, Pierre Marty, Laurent Boyer, Christelle Pomares

**Affiliations:** 1 Université Côte d’Azur, INSERM U1065, Centre Méditerranéen de Médecine Moléculaire (C3M) BP 23194 06204 Nice Cedex 3 France; 2 Service de Parasitologie-Mycologie, Centre Hospitalier Universitaire de Nice, Hôpital de l’Archet CS 23079 06202 Nice Cedex 3 France

**Keywords:** Treatments, Leishmaniases, Immunomodulator, Ethnopharmacology

## Abstract

Leishmaniases are vector-borne parasitic diseases that pose a threat to over 1 billion people worldwide. The parasites target cells of the reticulohistiocytic system, such as macrophages, where they replicate. The disease manifests in various forms, ranging from localized cutaneous leishmaniasis to life-threatening visceral forms, which are fatal in 95% of cases without treatment. Current treatments rely on the invasive administration of toxic and expensive drugs that are increasingly encountering resistance. Therefore, finding alternative treatments for this disease is imperative. This literature review focuses on recent advancements in alternative treatments and aims to present the various strategies designed to address current limitations, including cost, toxicity, off-target effects, administration routes, and the emergence of drug resistance. Starting with an overview of the existing approved treatments and their specific limitations, we categorize treatment development strategies into five key sections: (i) combination therapies using existing approved treatments to enhance efficacy and reduce resistance; (ii) nanoparticle formulations, which enable targeted delivery to infected organs and improved therapeutic efficiency; (iii) drug repositioning, a strategy that has already contributed to the approval of over half of current therapeutic compounds; (iv) immunomodulation, used in conjunction with standard chemotherapies to enhance treatment efficacy and lower relapse rates; and (v) ethnobotanicals, which have demonstrated promising *in vitro* results by combining low toxicity, immunomodulatory properties, and potent anti-parasitic effects. In summary, this review outlines current strategies in treatment development, emphasizing their advantages over conventional therapies while acknowledging their limitations.

## Abstract (fr)

Les leishmanioses sont des maladies parasitaires à transmission vectorielle qui menacent plus d’un milliard de personnes dans le monde. Les parasites ciblent les cellules du système réticulohistiocytaire, comme les macrophages, où ils se répliquent. La maladie se manifeste sous diverses formes, allant de la leishmaniose cutanée localisée aux formes viscérales potentiellement mortelles, qui sont responsables de décès dans 95% des cas sans traitement. Les traitements actuels reposent sur l’administration invasive de médicaments toxiques et coûteux qui rencontrent de plus en plus de résistances. Il est donc impératif de trouver des traitements alternatifs pour cette maladie. Cette revue de la littérature se concentre sur les avancées récentes en matière de traitements alternatifs et vise à présenter les différentes stratégies conçues pour remédier aux limitations actuelles, notamment le coût, la toxicité, les effets hors cible, les voies d’administration et l’émergence de la résistance aux médicaments. En commençant par un aperçu des traitements approuvés existants et de leurs limitations spécifiques, nous classons les stratégies de développement de traitements en cinq sections clés : (i) les thérapies combinées utilisant les traitements approuvés existants pour améliorer l’efficacité et réduire la résistance; (ii) les formulations de nanoparticules, qui permettent une administration ciblée aux organes infectés et une efficacité thérapeutique améliorée; (iii) le repositionnement des médicaments, une stratégie qui a déjà contribué à l’approbation de plus de la moitié des composés thérapeutiques actuels; (iv) l’immunomodulation, utilisée en conjonction avec les chimiothérapies standard pour améliorer l’efficacité du traitement et réduire les taux de rechute; (v) les produits ethnobotaniques, qui ont démontré des résultats *in vitro* prometteurs en combinant une faible toxicité, des propriétés immunomodulatrices et un puissant effet antiparasitaire. En résumé, cette revue décrit les stratégies actuelles de développement de traitements, en soulignant leurs avantages par rapport aux thérapies conventionnelles tout en reconnaissant leurs limites.

## Introduction

Leishmaniasis, a vector-borne disease transmitted through the bite of phlebotomine sand flies, poses a threat to more than 1 billion people in over 98 countries worldwide [111]. It is responsible for 20,000 to 30,000 deaths a year and has been considered an uncontrolled neglected tropical disease by the World Health Organization (WHO) since 2011 [111].

*Leishmania* parasites exhibit a preference for the reticulohistiocytic system in mammals, with macrophages serving as their primary host cells*.* Inside these cells, the parasite replicates until the macrophage ruptures, enabling the infection to spread. Over 20 species of *Leishmania* are known to cause disease, each exhibiting distinct tropisms that lead to outcomes ranging from asymptomatic carriage to diverse clinical manifestations. These manifestations range from self-healing skin ulcers to systemic infections characterized by hepatosplenomegaly, which can be fatal if left untreated.

Human leishmaniasis manifests in four main forms. The most common form, cutaneous leishmaniasis, causes ulcerative skin lesions and is typically associated with *Leishmania (L.) major, L. tropica*, or *L. mexicana.* Muco-cutaneous leishmaniasis (MCL) caused by *L. braziliensis, L. panamensis*, and *L. amazonensis* affects the mucous membranes of the nose and mouth, resulting in severe disfigurement as the disease progresses. Visceral leishmaniasis, the most severe form of the disease, primarily targets the liver and spleen, causing irregular fever and severe hepatosplenomegaly, with a mortality rate of 95% if left untreated. It is predominantly caused by *L. donovani*, which can also lead to post-kala-azar dermal leishmaniasis, a condition marked by widespread skin lesions that appear after an initial cure of visceral leishmaniasis [90]. *Leishmania infantum* is another species associated with visceral leishmaniasis.

The disease caused by *L. infantum* primarily affects dogs, which serve as the reservoir for this parasite. Non-infected sand flies can acquire the parasite during a blood meal from an infected dog and subsequently transmit it to other mammals, such as foxes, rodents, and humans. In dogs, clinical symptoms often begin with skin-related issues, such as alopecia, onychogryphosis, and eye problems including keratoconjunctivitis and uveitis. If untreated, these symptoms can progress to a visceral form, characterized by splenomegaly, kidney failure, weight loss, muscle wasting, and lethargy [77].

Currently, two vaccines are available for canine leishmaniasis caused by *L. infantum*: LetiFend^®^ in Europe, and Leish-Tec^®^ in Brazil [50]. However, no effective vaccine exists for humans [106]. Prevention in humans relies entirely on avoiding sand fly bites through the use of repellents, insecticide sprays, or mosquito nets. Once an infection occurs, treatment remains the only option. Pentavalent antimonials are commonly used as the first-line treatment in most countries. However, increasing drug resistance has become a major concern [36]. Nowadays, liposomal amphotericin B (L-AMB) is considered as the first-line treatment in high-income countries [80]. Despite their efficacy, existing treatments have significant limitations, including invasive administration, long treatment durations, high costs, and the emergence of resistance [75].

Given these challenges, there is an urgent need to develop novel alternative treatments. In this review, we discuss the limitations of current therapies and propose strategies to address these issues. Specifically, we focus on reducing toxicity, costs, and resistance, while exploring new administration routes and methods to shorten treatment durations.

## Materials and methods

This review concentrated on studies conducted *in vivo* and *in vitro* on amastigotes, as these are the intracellular forms responsible for the disease. Screening on promastigotes was excluded because, although amastigote-based screening offers a high hit-to-success ratio, promastigote-based screening often fails during later stages of drug development [23]. This review highlights strategies for developing treatments against leishmaniasis, focusing on progress made between 2015 and 2023. Bibliographic research was conducted on PubMed using keywords such as leishmaniasis, treatment, cutaneous or mucocutaneous or visceral, mechanism, and combination. Over 600 articles matching these keywords were identified and categorized based on the drug development strategy they addressed. First, we reviewed current therapies validated for treating *Leishmania*, along with their mechanism of action and limitations. Combination therapies were then explored, emphasizing the simultaneous use of several approved compounds. We also examined two key strategies responsible for more than half of the major approved treatments: nanoparticle carriers, as seen with liposomal amphotericin B, and drug repositioning exemplified by miltefosine and paromomycin. Additionally, we investigated articles on immunotherapy with current treatments and studies on ethnobotanics as a strategy for novel drug discovery.

## Results

### Current treatment options and limitations

**Figure 1:**
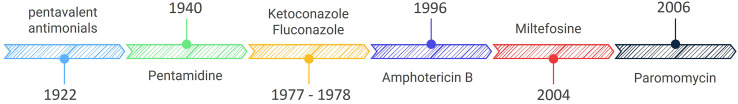
Current treatments and their development over time.

Currently, seven approved treatments are used against different forms of leishmaniasis worldwide (Table 1). Historically (Fig. 1), the first approved treatment, based on trivalent antimonials, was introduced in 1912. By 1922, these were replaced by safer pentavalent antimonials, leading to the development of today’s two approved molecules: sodium stibogluconate (SSG) and meglumine antimoniate. These drugs were highly effective until the 1970s and 1980s, when significant treatment failures and the emergence of resistant strains began to be observed [110]. Despite their efficacy, they are associated with side effects such as gastrointestinal issues (vomiting, anorexia) and cardiotoxicity. Pentamidine, approved in the 1940s, is another drug used to treat leishmaniasis. However, its use has been restricted by the WHO due to severe side effects, including diabetes mellitus, severe hypoglycemia, shock, and myocarditis. Ketoconazole and fluconazole, discovered in 1977 and 1978, respectively are treatment options for cutaneous leishmaniasis [110]. However, the development of resistance in *Leishmania* parasites has limited their effectiveness. In 1996, the approval of amphotericin B (AMB) addressed some resistance issues, but this drug is associated with side effects such as fever, chills, rigor, and nephrotoxicity. In 1999, L-AMB was developed to reduce toxicity. While liposomal encapsulation improved safety, it significantly increased the cost, making it less accessible in developing countries [12]. Until 2004, all treatments for visceral leishmaniasis required invasive administration via the intravenous or intramuscular routes. This made treatment difficult in some regions, where multiple hospital visits were required for injections. The approval of miltefosine in 2004 marked a breakthrough as the first oral drug for leishmaniasis. Despite its advantages, miltefosine is teratogenic, limiting its use in pregnant women, and causes gastrointestinal side effects [12]. Paromomycin, an aminoside antibiotic, became the most recent anti-leishmanial drug approved in 2006 [16, 86]. However, its use is limited due to side effects, including ototoxicity, renal toxicity, and hepatotoxicity.

**Table 1:** Classical monotherapies used for *Leishmania* infection.

Compound	Commercial name	Average cost in US$ [4, 14, 57]	Route of administration	Mechanism of action	References
Sodium stibogluconate (SSG)	Pentostam^®^	Cost of VL treatment in Sudan: 450	IM/IV	Unclear, but can lead to inhibition of DNA topoisomerase I and increased ROS production. To date it remains unknown if it is the pentavalent form or the reduced trivalent form exerting leishmanicidal activity.	[28]
Meglumine antimoniate	Glucantime^®^	Cost of VL treatment in Brazil IM: 418.52/IV 669.40 Cost of mucosal leishmaniasis treatment in Brazil 167.66	IM/IV/IL	Unclear, but induces DNA damage mediated by oxidative stress and glutathione (GSH) depletion.	[61]
Amphotericin B deoxycholate	Fungizone^®^	Cost of VL treatment in Brazil 1522.70	IV	Interaction with membrane ergosterol, pore formation, and leakage of cytosolic content. Sequestration of cholesterol abrogating parasite/macrophage interaction. Functional gene overexpression leading to ROS and Ca^2+^ increase, cytochrome-c liberation and metacaspase activation, inducing DNA fragmentation and apoptosis. Immunomodulation of chemokines and cytokines, including IL-1B, leading to increasing NO levels.	[12]
Liposomal Amphotericin B	AmBisome^®^	Cost of VL treatment in Brazil 659.79 to 11,559.15Cost of mucosal leishmaniasis treatment in Brazil: 715.35In South-East Asia and East Africa: Donation of the drug for VL treatment from Gilead Science	IV		
Miltefosine	Impavido^®^	Cost of mucosal leishmaniasis treatment in Brazil: 259.92	O	Inhibition of phosphatidylcholine and sphingomyelin biosynthesis leading to apoptosis. Disruption of Ca^2+^ homeostasis, alkalinization of acidocalcisomes. Induction of IFN-g, leading to a Th1 inflammatory profile. Respiratory chain disruption by cytochrome-c inhibition.	[12]
Paromomycin	Humatin^®^		IM	Affects *Leishmania* cytosolic RNA translation and intracellular trafficking by inhibition of protein synthesis and targeting the decoding center of ribosomes.	[16, 86]
Pentamidine	Pentacarinat^®^		IM/IV	Unclear, but other diamine compounds exert their activity by accumulating and blocking the replication of *Leishmania* kinetoplast DNA.	[113]

IM: intramuscular; IV: intravenous; IL: intralesional, O: oral.

Today, these drugs (Table 1) remain the first-line treatments recommended by WHO guidelines for all forms of leishmaniasis. Despite their efficacy, they have significant limitations. Most treatments require invasive administration, have long treatment durations, and are associated with high costs and toxicity. Additionally, treatment failures are becoming more frequent, and resistant *Leishmania* isolates are increasingly reported for all approved regimens [75].

### Combination therapies

Combination therapy is a promising strategy to address the limitations of current anti-leishmanial drugs. By combining two treatments with complementary or distinct mechanisms of action, this approach seeks to enhance therapeutic efficacy and potentially achieve synergistic effects. Targeting different biological pathways or stages of the parasite’s lifecycle also reduces the likelihood of developing drug resistance. For instance, Mutiso et al*.* demonstrated the effectiveness of combining diminazene and artesunate—two drugs with distinct mechanisms of action against *L. donovani* in a mouse model [66]. This combination was more effective than either drug alone, highlighting the potential for synergistic effects. It also reduced drug doses, lowering toxicity and treatment costs, particularly for expensive therapies like L-AMB [66].

Several drug combinations have been tested with success, significantly reducing treatment durations (Tables 2 and 3). In humans, the combination of 20 mg/kg SSG and 15 mg/kg paromomycin reduced treatment time from 30 days to 17 days, maintaining the same efficacy. In India, combining L-AMB with 50–100 mg/kg miltefosine or 11 mg/kg paromomycin shortened treatment duration from 31 to 7–10 days. It also reduced the L-AMB dose from 15 mg/kg over 30 days to a single 5 mg/kg injection, decreasing cost and toxicity [38, 88]. To select an appropriate treatment, one approach is to align with the WHO recommendations [110, 112]. For HIV-positive patients, a combination of 30 mg/kg L-AMB and 100 mg/day miltefosine over 25 days increased cure rates from 50% with L-AMB monotherapy (40 mg/kg) to 81% [105, 112].

**Table 2:** Combination therapies for human leishmaniases.

Combination treatments		Advantages	Region	*Leishmania* species	References
		**Human VL**			
SSG (IV/IM, 20 mg/kg, 17 d)	Paromomycin (IM, 15 mg/kg, 17 days)	Treatment reduction time from 30 to 17 days. Reduced hospitalization.	Eastern Africa	*L. donovani* and *L. infantum*	[37]
L-AMB (IV, 5 mg/kg, single dose)	Miltefosine (O, 50–100 mg/day, 7 days) or paromomycin (IM, 11 mg/kg/day, 10 days)	Reduction from 15 mg/kg total over 30 days to a single injection of 5 mg/kg. Fewer adverse events and reduction of treatment time (31 to 15 days).	India		[37]
Miltefosine (O, 50–100 mg/day, 10 d)	Paromomycin (IM, 11 mg/kg/day, 10 days)				
L-AMB (IV, 10 mg/kg, single dose)	SSG (IM, 20 mg/kg/day, 10 d) or miltefosine (O, 2.5 mg/kg/day, 10 days)	Efficacy <90% cure in phase II, no phase III trial.	Eastern Africa	*L. donovani*	[109]
L-AMB (IV, 30 mg/kg total, 5 mg/kg/d on alternate days)	Miltefosine (O, 100 mg/day, 28 days)	Increased efficacy compared to monotherapy at 40 mg/kg as recommended by the WHO; highest documented efficacy in HIV+/VL patients.	Ethiopia		[26]
		**Human *L. braziliensis***			
Miltefosine (O, 150 mg/day, 28 days)	Pentamidine (IL, 120 μg/mm^2^ lesion area on alternate days for 3 days)	Additive effect, fewer adverse effects, and reduced cost. Alternative for local treatment, dissemination prevention, or when avoiding parenteral administration.	Bolivia	*L. braziliensis*	[93]
		**Human CL**			
Glucantime (IL, 1 mL/cm^2^ of lesion, 1×/week, 6 weeks)	Itraconazole (O, 200 mg/day, 6 weeks)	No benefit on efficacy or treatment duration compared to Glucantime^®^ alone.	Pakistan	Cutaneous leishmaniasis	[8]

IV: intravenous; IM: intramuscular O: oral; IL: intralesional.

**Table 3:** Combination therapies for canine leishmaniases.

Combination treatments		Advantages	Region	*Leishmania* species	References
Meglumine antimoniate (SC, 100 mg/kg/day, 28 days) or paromomycin (SC, 15 mg/kg/day, 28 days)	Allopurinol (O, 10 mg/kg 2×/day, 2 months)	This study aimed to prove the efficacy and safety of the paromomycin/allopurinol combination in non-azotemic dogs and compared it to the reference treatment meglumine antimoniate/allopurinol	Greece	*L. infantum*	[44]
Meglumine antimoniate (SC, 100 mg/kg/day, 30 days) or miltefosine (O, 2 mg/kg/day, 30 days)	Allopurinol (O, 10 mg/kg 2×/day, 2 months)Allopurinol (O, 10 mg/kg/day, 30 days then maintained over 6 years)	Both therapies show a similar efficacy in parasite burden reduction. Fewer relapses were observed in combination with meglumine antimoniate compared to miltefosine	Italy		[54]
Meglumine antimoniate (SC, 100 mg/kg/day, 4 weeks) or miltefosine (O, 2 mg/kg/day, 4 weeks)	Allopurinol (O, 10 mg/kg 2×/day, 6 months)	Both treatment protocols favor remission. Both combination treatments lead to a sustained inflammatory environment deleterious to the parasite, directly affecting cytokine generation	Portugal		[84]

O: oral; SC: subcutaneous.

In dogs, reference treatment is a combination therapy of 100 mg/kg/day meglumine antimoniate and allopurinol or miltefosine and allopurinol [50]. Miltefosine promotes remission by sustaining the inflammatory environment harmful to the parasite [84]. However, Manna et al*.* reported fewer relapses with meglumine antimoniate and allopurinol compared to miltefosine alone [54]. Paromomycin showed safety in dogs, but did not significantly improve cure rates compared to the reference treatment [44].

Experimental treatments in hamsters have explored topical applications of paromomycin for cutaneous leishmaniasis in combination with meglumine antimoniate (Glucantime^®^) or miltefosine. These combinations, even at suboptimal doses, were more effective than monotherapy, suggesting that topical paromomycin may be a viable option for reducing parasite load [60]. Combining miltefosine and paromomycin also showed enhanced efficacy, with no observed cross-resistance development upon repeated exposure [42].

While combination therapies can improve efficacy, reduce toxicity, and lower costs, they are not always successful [8, 93, 109]. Risks include the potential amplification of the side effects of both treatments and the potential development of drug resistance. For example, *L. donovani* has shown resistance to certain drug combinations *in vitro*, as reported by García-Hernández et al*.* [31]. These findings highlight the need for careful monitoring and the strategic use of combination therapies to mitigate the risk of resistance. In addition to combination therapies, other strategies, such as improving drug delivery systems using nanoparticles, have shown promise. Encapsulation of anti-leishmanial treatments, such as amphotericin B, has demonstrated potential to enhance efficacy and reduce limitations.

### Nanoparticle carriers

Nanoparticles are small assemblies of organic or inorganic matter, sized between 1 and 100 nm, that offer significant potential in drug delivery. Organic nanoparticles can be classified into two main subgroups: polymeric nanoparticles, such as dendrimers, and lipid-based nanoparticles, such as liposomes. Lipid-based nanoparticles, primarily composed of phospholipids, are highly biocompatible and assemble into bilayered structures due to their amphipathic properties. These structures have a hydrophobic layer and a hydrophilic interior, enabling the solubilization and delivery of both hydrophilic and hydrophobic drugs. Among nanoparticle types, liposomes are the most commonly FDA-approved for clinical use. Polymeric nanoparticles offer greater structural variability due to their customizable compositions, allowing for the insertion of compounds to target specific intracellular sites [59]. Nanoparticles have largely been studied to improve the efficacy of anti-cancer drugs [115]. In anti-leishmanial therapy, liposomes have already demonstrated effectiveness, as amphotericin B is clinically administered in its liposomal form. Beyond liposomal formulations, several approved anti-leishmanial treatments have been encapsulated in various nanoparticles and tested in experimental models, such as mice and hamsters, to improve drug efficacy (Tables 4 and 5). Encapsulating approved treatments in lipid-based nanoparticles, such as liposomes, enhances drug stability and protects them from degradation in harsh environments, such as gastrointestinal fluids [29]. This allows for oral administration of drugs such as AMB [76], paromomycin [2], and pentamidine [102]. Additionally, nanoparticles enable topical administration of AMB, meglumine antimoniate, miltefosine [100], and SSG [20]. Liposomal formulations provide controlled drug release, extending the drug’s half-life and reducing peak concentrations, thereby lowering toxicity, particularly on renal function. Liposomes, being lipid-based, naturally interact with macrophage-rich organs such as the liver and spleen, improving bioavailability while minimizing systemic toxicity [89]. Nanoparticles can be functionalized with targeting groups to direct drugs to specific cells or organs. For example, phosphatidylserine signals macrophages, targeting the liver and spleen [89], lactoferrin interacts with C-type lectin receptors on antigen-presenting cells [6], guar gum targets mannose-like receptors on macrophages [76] and chitosan promotes phagocytosis, favoring liver and spleen targeting [101].

**Table 4:** Nanoparticle carriers with amphotericin B.

Type of nanoparticle/Main components	*Leishmania* species	Administration/host species	Effect compared to drug’s free form	References
PN/PLGA	*L. major*	IL/mouse	Efficacy increased in IL injection with no systemic toxicity.	[1]
PN/PLGA – PS	*L. donovani*	IV/hamster	Increased efficacy through specific distribution (liver, spleen).	[89]
PN/PLGA – lactoferrin	*L. donovani*	IP/hamster	Efficacy increased by accumulation in liver and spleen, reduced toxicity.	[5]
PN/PLGA – PEG	*L. donovani*	IV/hamster	Increased efficacy compared to free form.	[48]
PN/PLGA – stearylamine	*L. donovani*	IP/hamster	Toxicity decreased. Promote a Th1 response. Synergistic effect of the drug with stearylamine.	[6]
PN/BSA	*L. amazonensis*	IP/mouse	Toxicity decreased. Superior efficacy towards amastigotes.	[15]
PN/Glycol chitosan stearate	*L. donovani*	IP/hamster	Toxicity decreased. Efficacy increased. Specific distribution (liver, spleen) and less in kidneys.	[40]
PN/Chitosan anchor and miltefosine stabilization	*L. donovani*	IP/hamster	Toxicity decreased. Specific distribution to target organs (liver, spleen).	[100]
PN/TGNP	*L. amazonensis*	IP/hamster	Increased efficacy. Reduced toxicity.	[96]
PN/Guar gum – Eudragit – Piperine	*L. donovani*	O/IP/hamster	Toxicity decreased. Efficacy increased. Specific distribution (liver, spleen). Less nephrotoxicity. Increased activity upon oral delivery.	[76]
PN or dendrimer/Chitosan nanoparticles or LGD	*L. major*	IP/mouse	Toxicity decreased and efficacy increased.	[114]
Dendrimer/ALGD	*L. major*	IP/mouse	Increased efficacy and solubility. Toxicity decreased.	[32]
Liposome – polymer/DSHemsPC	*L. major*	IV/mouse	Cost decreased. Same efficacy.	[43]
Liposome/PC – Cholesterol	*L. major*	T/mouse	Increased efficacy due to higher penetration properties.	[100]
Solid lipid nanoparticle/Compritol^®^ 888 ATO	*L. major*	T/mouse	Increased efficacy, reduction in lesion size and amastigote count.	[91]
PN/Polycaprolactone	*L. amazonensis* or *L. infantum*	RO/mouse	Increased specificity in targeting to liver, spleen and lungs.	[94]
Liposome – polymer/Stearylamine	*L. major*	T/mouse	Direct activity of stearylamine. Increased permeation of the cream.	[100]
Liposome/Cholesterol – DP – DSPC and DSPE – PEG2000	*L. infantum*	IV/mouse	Immunomodulatory effect in favor of a Th1 response with reduction of inflammation.	[78]

IV: intravenous; IP: intraperitoneal; IL: intralesional; O: oral; T: topical; RO: retro-orbital; PN: polymeric nanoparticle; PLGA: poly-lactic-co-glycolic acid; LGD: linear globular dendrimers; ALGD: anionic linear globular dendrimer; BSA: bovine serum albumin; PEG: polyethylene glycol; PC: phosphatidylcholine; PS: phosphaditylserine; DSPC: distearoylphosphatidylcholine; DSPE: distearoylphosphatidylethanolamine; DP: dicetylphosphate; DSHemsPC: 1,2-distigmasterylhemisuccinoyl-sn-glycero-3-phosphocholine; TGNP: triglyceride-rich nanoparticles. Mouse model used in articles is BALB/c mice. Hamster model used in articles is Syrian Golden Hamsters.

**Table 5:** Nanoparticle carriers with miltefosine, pentamidine or SSG.

Type of nanoparticle/Main components	*Leishmania* species	Administration/host species	Effect compared to drug’s free form	References
**Miltefosine**				
Polymeric nanoparticle/PLGA-PEG with CD14	*L. donovani*	IV/hamster	Efficacy increased with a decrease of EC_50_ due to specific macrophage targeting.	[49]
Liposome/PC – Cholesterol	*L. infantum*	O/mouse	Toxicity decreased (macrophages, gastrointestinal irritability) due to oral administration and increased stability.	[29]
Liposome/PC – Cholesterol	*L. major*	T/mouse	Limitation of systemic toxicity by topical application.	[100]
Polymeric nanoparticle/PLGA – Mannosylated thiolated chitosan	*L. donovani*	O/mouse	Efficacy increased (decreased IC50) due to high tissue permeation, with decrease toxicity.	[2]
Liposome/PC – PEG	*L. infantum*	IV/mouse	Increased efficacy by targeting the spleen, liver, and lungs. Increased persistence in blood through stabilization.	[32]
Solid lipid nanoparticle/Stearic acid	*L. major*	IM/mouse	Efficacy increased with immunomodulatory effects towards a Th1 response.	[41]
**Pentamidine**				
Polymeric nanoparticle/PLGA	*L. infantum*	O/mouse	Facilitated administration by oral use.	[102]
**SSG**				
Liposome/Phospholipon^®^	*L. tropica*	T/mouse	Increased efficacy (decreased IC_50_ and increased selectivity index). Better retention in deep skin layers without permeation enhancers.	[19]

IV: intravenous; IM: intramuscular; O: oral; T: topical; PLGA: poly-lactic-co-glycolic acid; PEG: polyethylene glycol; PC: phosphatidylcholine. EC50: half-maximal effective concentration, IC50: half-maximal inhibitory concentration. Mouse model used in articles is BALB/c mice. Hamster model used in articles is Syrian Golden Hamsters.

Polymeric structures like dendrimers significantly increase the solubility of hydrophobic compounds. For instance, dendrimer formulations increased amphotericin B solubility by 478-fold using an anionic linear globular dendrimer carrier [32]. Some nanoparticles have immunomodulatory properties. Stearylamine-based lipid nanoparticles without any drug increased anti-leishmanial activity by promoting a Th1 immune response, upregulating IL-12, IFN-γ, and TNF-α, and activating iNOS pathways [5]. Similarly, compounds like lactoferrin and guar gum can activate macrophages, stimulating innate immune responses [40, 76].

In addition to encapsulating approved treatments, drug repositioning remains a viable strategy for addressing leishmaniasis. Repositioned drugs, such as amphotericin B, miltefosine, and paromomycin are already used as first-line treatments in many countries.

### Drug repositioning

Approximately 60% of currently approved anti-leishmanial drugs are derived from the repositioning of existing medications initially developed for diseases other than leishmaniasis [12].

The most prominent class of repositioned drugs includes antimicrobial compounds (Table 6). For instance, amphotericin B is an anti-fungal that has been successfully repositioned for visceral leishmaniasis. Other anti-fungals, such as ketoconazole, fluconazole, and itraconazole, are recommended by WHO for cutaneous leishmaniasis [110]. These drugs target the ergosterol pathway, which is essential for the cell membranes of *Leishmania*, offering high selectivity without affecting mammalian cells. Promising candidates like butenafine, ravuconazole, and miconazole have also demonstrated their efficacy [3, 9, 99]. Paromomycin, an antibiotic belonging to the aminoglycoside class, can be used for cutaneous leishmaniasis (CL) as a topical treatment. Clinical trials showed that paromomycin cream achieved cure rates close to 80%, providing a non-invasive and effective alternative for CL [92]. Similarly, delamanid and SQ109, originally developed for tuberculosis have shown efficacy against *L. donovani.* Delamanid offers the added advantage of oral administration [33, 74].

**Table 6:** Repositioned drugs and their mechanisms of action.

Name of the compound	Function	Mechanism of action	References
**Anti-fungal**			
Miconazole	Anti-fungal	Disruption of sterol synthesis.	[3]
Ravuconazole	Invasive fungal infections	Azoles inhibit the sterol biosynthesis pathway by inhibiting the conversion of lanosterol to zymosterol by the monooxygenase lanosterol C14α-demethylase.	[99]
Butenafine	Fungal skin infections (ringworm, athlete’s foot, jock itch, pityriasis)	Ergosterol biosynthesis disruption via inhibition of squalene epoxidase.	[10]
**Anti-bacterial**			
Delamanid	Anti-tuberculosis (approved for multidrug resistant strains)	Inhibition of mycobacterial cell wall components synthesis, methoxy mycolic acid and ketomycolic acid. Activation by the enzyme deazaflavin-dependent nitroreductase, leading to a reactive intermediate metabolite that inhibits mycolic acid production.	[74]
SQ109	Resistant mycobacterium tuberculosis (phase IIb/III)	Disruption of intracellular Ca^2+^ homeostasis, collapsing of the mitochondrial electrochemical potential and affecting acidocalcisomes.	[33]
**Anti-trypanosomatids**			
Fexinidazole	Anti-trypanosomiasis (clinical trial phase II/III in 2019)	Targets nitroreductase in trypanosomatids, which possesses a homolog in *Leishmania* parasites.	[74]
Suramin	Hemolymphatic stage of African trypanosomiasis (*Trypanosoma. brucei rhodesiense*)	Inhibition of glycolytic enzymes of the parasite. Elevates pro-inflammatory Th1 cytokine secretion while suppressing Th2 responses.	[46]
**Anti-cancer**			
Miransertib (ARQ 092)	PI3K/Akt-driven tumors or Proteus syndrome	Akt inhibitor, activated by *Leishmania* and regulates cell growth, survival, and metabolism by phosphorylating downstream targets.	[67]
AR-12 (OSU-03012)	Anti-cancer (FDA IND-approved)	Host-mediated compound promoting other intracellular pathogen eradication, mediated by regulation of autophagy and Akt kinase pathway inhibition.	[17]
Ibrutinib	Anti-cancer for B cell malignancy	ITK/BTK inhibitor, blocking B-cell receptor signaling and proliferation (activated by *Leishmania*). Modulation of T-helper response.	[104]
EAPB0503 (Imiquimod analog) and Imiquimod	Skin cancer and condyloma	TLR-7 agonist leading to NF-kB pathway activation.	[27]
**Anti-depressants**			
Sertraline	Anti-depressant	Serotonin reuptake inhibitor	[52]
Clomipramine	Anti-depressant and anxiolytic, treatment of psychiatric disorders and OCD	Selective inhibition of serotonin-reuptake. Previous repurposing studies showed effect on parasites through trypanothione reductase.	[87]
Imipramine	Severe chronic depression	Inhibition of serotonin and norepinephrine reuptake. Known interaction with lipid bilayers and inhibition of methyltransferases leading to membrane disruption.	[3]
**Other**			
Simvastatin	Cholesterol reducer	Increase in LDL-cholesterol degradation and HMG-CoA reductase inhibitor.	[72]
Rapamycin, GSK-2126458	Graft rejection prevention Immunosuppressor	mTOR inhibitors. TOR from *Leishmania* is important in autophagy, and TOR1 and 2 are essential to parasite growth and virulence.	[45]
Amiodarone	Antiarrhythmic	Disruption of intracellular Ca^2+^ homeostasis by direct action on mitochondrion and acidocalcisomes. Blocking of sterol biosynthesis pathway through inhibition of squalene epoxidase activity.	[71]

ITK/BTK: interleukin 2-inducible T-cell kinase/Bruton tyrosine kinase; HMG-CoA: hydroxymethylglutaryl-CoA reductase; mTRO: mechanistic target of rapamycin; OCD: obsessive-compulsive disorder; TLR: toll-like receptor.

Other antimicrobial repositioned drugs, such as fexinidazole and suramin, target metabolic pathways in *Leishmania*. Fexinidazole showed efficacy comparable to approved drugs. Indeed, 200 mg/kg of fexinidazole showed activity similar to the approved drugs miltefosine and SSG (Pentostam^®^). Suramin, with its immunomodulatory properties, reduced parasite burden in a mouse model infected by *L. donovani* [46, 74].

Anti-cancer drugs like miltefosine, ibrutinib, and imiquimod enhance host-mediated responses, particularly pro-inflammatory cytokine production [27, 103]. Miransertib and AR-12 reduced parasite burdens *in vivo*. Surprisingly, anti-depressants like sertraline and clomipramine have also shown potential by disrupting parasite mitochondrial functions and metabolic pathways. These drugs induced oxidative stress, ATP depletion, and apoptosis in *Leishmania* [52, 87].

Cholesterol reducer simvastatin boosts host defense by enhancing phagosome maturation, while rapamycin biases immune responses toward protective Th1 cytokines. The anti-arrhythmic drug amiodarone disrupts calcium homeostasis and sterol biosynthesis in *Leishmania* parasites [46, 71, 72].

Repositioning drugs not only leverages existing treatments, but also provides faster and more cost-effective solutions for combating leishmaniasis. However, to date, most assays using repositioned drugs against *Leishmania* have been conducted *in vitro*, with some also tested in mouse models. Many steps remain before these drugs can be used to treat patients. Combining immunomodulators with conventional treatments could further enhance their efficacy (Table 6).

### Immunotherapies

**Figure 2:**
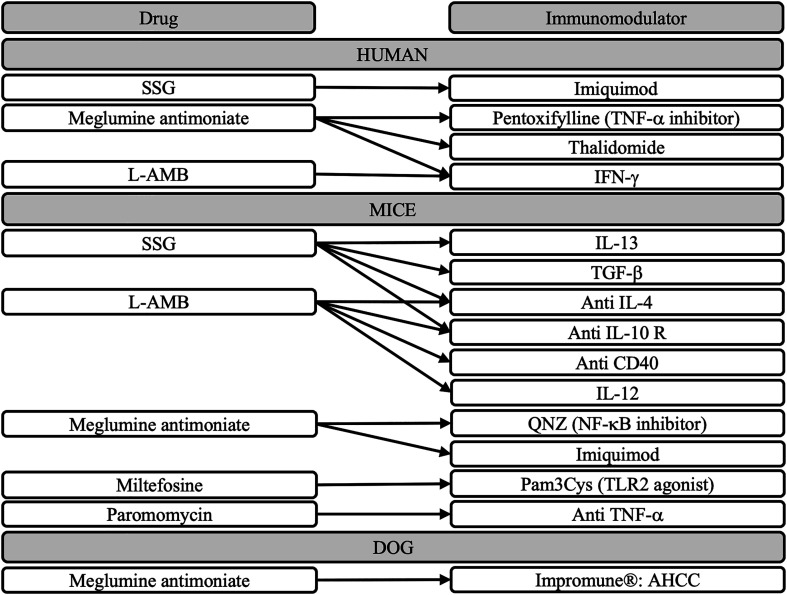
Immunochemotherapy tested in combination with drugs against *Leishmania* [9, 13, 35, 47, 53, 58, 63, 66, 85, 98, 107].

Over the last 20 years, immunotherapy has been developed either as monotherapy or in combination with classically used treatments for leishmaniasis (Fig. 2). Immunity plays a critical role in the progression and outcome of the disease. Generally, Th1 immune responses involving IL-12, IL-18, IFN-g, and TNF-a are considered protective cytokines in *Leishmania* infections. Conversely, Th2 responses dominated by IL-4, IL-10, IL-13, and TGF-b favored parasite survival and disease progression [108]. For many years, research on leishmaniasis revolved around this Th1/Th2 dichotomy. However, it is now accepted that controlling *Leishmania* efficiently requires coordinated action of both pathways. Additionally, susceptibility to leishmaniasis depends on both host immunity and the virulence of the parasite species [38]. Combining current treatments with immunotherapy could reduce the concentration and duration of treatments for leishmaniasis, while shifting host immunity to favor *Leishmania* control.

Since Th1 responses are protective against *Leishmania* infections, the first strategy used to enhance approved treatments has been to stimulate associated cytokines to favor remission. In dog models, domperidone and IFN-γ favor Th1 immune response and demonstrated activities against *Leishmania*. Domperidone is used for both prevention and treatment of canine leishmaniasis due to *L. infantum* [9]. Additionally, combining Glucantime^®^ with IFN-γ has been shown to improve cure rates and reduce treatment duration, whereas Glucantime^®^ plus active hexose correlated compound (AHCC) reduced adverse effects caused by allopurinol [9]. In mice, administering IL-12 with AMB increased the efficacy of a 2 mg/kg dose to match that of a 15 mg/kg dose [64].

Agonists of toll-like receptors (TLRs) have also been proposed. These receptors recognize pathogen-associated molecular patterns (PAMPs) [68] and trigger cytokine release via NF-κB activation, promoting pro-inflammatory responses. For example, the TLR-7 agonist imiquimod increased the efficacy of Glucantime^®^ against *L. major* [98]*.* Similarly, the TLR-2 agonist Pam3Cys enhanced the efficacy of suboptimal doses of oral miltefosine against *L. donovani* in a mouse model [9].

Another strategy involves inhibiting Th2 responses by modulating cytokines. In mice, anti-IL-10R treatment accelerated *L. donovani* elimination and increased efficacy when combined with either Pentostam^®^ or AMB [63–65].

In humans, the combination of IFN-γ with L-AMB has proven effective in curing a multi-resistant strain [47]. Thalidomide, an immunomodulator, reduced TNF-α secretion while increasing IFN-γ levels, thereby enhancing Th1 responses against a multi-resistant strain of mucosal leishmaniasis [35]. Despite the promising results of immunotherapies in humans, studies to date have been limited to small series or individual case reports.

Although immunotherapy is a relatively recent field, it shows promise but requires complex experimentation and development. Research into plant extracts has also emerged as a potential method to shift host immune responses favorably for *Leishmania* elimination*.*

### Ethnopharmacology

Ethnopharmacology is a rapidly growing scientific field that integrates botany, chemistry, and pharmacology to evaluate the biological activity and scientific validity of plants traditionally used in medicine. By leveraging the knowledge of indigenous populations, this approach helps identify plants with potential for drug development. It can also save time by serving as a shortcut for discovering new active compounds.

All the compounds discussed in the *in vivo* studies (Table 7) were tested directly against *Leishmania* infections. These studies demonstrated reductions in parasite burden, often attributed to an increase in Th1 immune response and a decrease in Th2 cytokine secretion, thereby promoting host survival over parasite persistence. Several compounds have been specifically studied for their effects on *Leishmania* parasite clearance. Lupeol, mahanine, and extracts from *Croton caudatus* Geisel have been shown to upregulate reactive oxygen species (ROS) and nitric oxide (NO) pathways, both of which are critical for destroying intracellular pathogens [22, 25, 81]. Molecular docking identified specific parasite targets for some compounds. Lupeol, for example, bound to key *Leishmania* proteins such as PTR1, APRT, LPG, and GP63 [22]. *Croton caudatus* Geisel extract targets ascorbate peroxidase, a parasite antioxidant [25]. These finding suggested dual mechanisms, direct parasite targeting, and host immune modulation. In addition to their potential dual mechanisms, oral formulation of mahanine and JdHex offered easier administration compared to traditional anti-leishmanial drugs [25, 81].

**Table 7:** Ethnopharmacology.

Plant	Family	Compounds (major/active)	*Leishmania* species	Effect	References
***In vivo* mice (BALB/c)**					
*Sterculia villosa*	Malvaceae	Lupeol	*L. donovani*	*In vitro*, activity against promastigote and amastigote forms with an increase of NO. *In vivo*, 75 mg/kg/day treatment reduced splenic and hepatic burden and up regulated the release of pro inflammatory Th1 cytokines IL-12 and IFN-g, while down regulating release of anti-inflammatory IL-10 and TGF-b. Molecular docking revealed binding to 4 major potential drug targets (PTR1, APRT, biosynthetic LPG, and GP63).	[22]
*Croton caudatus* Geisel. (var. tomentosus Hook)	Euphorbiaceae	Terpenoids/semi-purified hexane extract of *C. caudatus* leaves (JdHex)	*L. donovani*	*In vitro*, alteration of promastigote metabolism (lipids, proteins, carbohydrates) and integrity (DNA condensation, PS externalization, apoptosis). Reduced replication of amastigotes, increased release of NO, pro-inflammatory IL-12 and TNF-alpha, reduction of TGF-beta and IL-10. *In vivo*, reduction of parasite burden in liver and spleen, induction of Th1 response by IFN-g secretion and abrogation of IL-10 secretion.	[25]
*Murraya koenigii*	Rutaceae	Mahanine	*L. donovani*	*In vitro*, apoptosis through phosphatidylserine externalization. Increased ROS and NO generation, suppression of Uncoupling protein 2 and Th1 cytokines through modulation of the STAT pathway. Molecular modeling revealed interaction with parasite antioxidant enzymes like ascorbate peroxidase. *In vivo*, reduction of parasite burden, upregulation of NO, iNOS, ROS, IL-12, and T cell proliferation	[81]
*Pentalinon andrieuxii*	Apocynaceae	Pentalinonsterol	*L. donovani*	*In vivo*, targeted towards infected organs and reduction of parasite load in liver, spleen and bone marrow. Enhanced T cell proliferation. Strong Th1 protective response with enhanced IFN-g production and formation of mature hepatic granulomas. No modulation of anti-inflammatory cytokines.	[39]
*Bursera aptera*	Burseraceae	Podophyllotoxin	*L. mexicana*	*In vitro*, promastigote apoptosis and decreased mitochondrial membrane potential. *In vivo*, reduction in lesion size and parasite burden. Increased Th1 cytokines TNF-α and IFN-g, and decreased Th2 cytokines IL-4 and IL-10 in sera of mice.	[69]
**Intracellular amastigotes**					
*Rhynchostylis retusa*, *Tropidia curculioides*, *Satyrium nepalense*	Orchidaceae	NA	*L. donovani*	*Rhynchostylis retusa* root extract was active against intracellular amastigotes with low cytotoxicity.	[11]
*Physalis angulata*	Solanaceae	NA	*L. amazonensis*	Aqueous extract of *P. angulata* (AEPa) root increased ROS which induced *Leishmania* cell death by apoptosis. AEPa increased macrophage activation state and promoted synthesis of superoxide anion (O_2_^−^).	[18]
*Euterpe oleracea* “Açai”	Arecaceae	Anthocyanins, phenolic compounds	*L. amazonensis*/*L. infantum*	Clarified Açai juice increased ROS levels and externalization of PS marking apoptosis. Reduce amastigote load inside cells for *L. amazonensis* and *L. infantum*. Led to strong reduction in IL-17 levels in infected cells.	[19]
*Tetradenia riparia* (Hochstetter) Codd	Lamiaceae	NA	*L. amazonensis*	Reversion of parasite mediated inhibition of IFN-gamma secretion, blocking of induction of IL-10, IL-4, and IL-5, and inhibition of secretion of IL-1B, IL-17, IL-33, and TNF-α.	[24]
*Croton cajucara* Benth. “sacaca”	Euphorbiaceae	Trans-dehydrocrotonin (DCTN), trans-crotonin (CTN) and acetylaleuritolic acid (AAA)	*L. amazonensis*	Inhibition of trypanothione reductase enzyme.	[51]
*Stachytarpheta cayennensis* (Rich.) Vahl.	Verbenaceae	Verbascoside, isoverbascoside (ratio 7:3)	*L .amazonensis*	Selective inhibition of parasite arginase.	[55]
*Zingiber zerumbet* (L.) Smith (Shampoo ginger)	Zingiberaceae	Zerumbone	*L. donovani*	Increased ROS, led to DNA condensation and phosphatidylserine externalization followed by apoptosis.	[62]
*Syzygium cumini* (L.) Skeels “jambolão”	Myrtaceae	α-pinene	*L. amazonensis*	Immunomodulatory activity by increase of NO secretion and phagocytic and lysosomal activity.	[79]
*Platonia insignis* Mart. “bacurizeiro”	Clusiaceae	Lupeol	*L. amazonensis*	Led to increased lysosomal volume and phagocytic capacity of macrophages.	[95]
46 plants	Varying	NA	*L. donovani*	Of the 46 plants, 15 extracts showed activity against *Leishmania* parasites.	[104]
*Stachytarpheta cayennensis*	Verbenaceae	Verbascoside	*L. amazonensis*	Inhibition of parasite arginase, leading to reduced protective oxidative mechanisms with impaired trypanothione synthesis.	[56]
*Alternanthera brasiliana* (L.) Kuntze, *Eugenia uniflora* L., *Jatropha gossypiifolia*, *Schinus terebinthifolia* Raddi	Amaranthaceae, Myrtaceae, Euphorbiaceae, Anacardiaceae	Tirucallane type triterpenoids schinol and masticadienoic acid (*S. terebinthifolia*), sesquiterpene atractylon, glucosylated flavonoids including quercitrin (*E. uniflora*)	*L. amazonensis*	*E. uniflora* extracts contained quercitrin already reported as an arginase inhibitor.	[83]
**Axenic amastigotes**					
*Ajuga laxmannii*	Lamiaceae	Harpagide, 8-O-acetylharpagide, cis-melilotoside, trans-melilotoside, dihydromelilotoside, verbascoside, galactosylmartynoside, isoorientin.	*L. donovani*	The iridoid glucoside 8-O-acetylharpagide, 8-O-acetylharpagide, and verbascoside were the most active against *L. donovani.*	[7]
45 plants	Varying	NA	*L. donovani*	Study revealed over 80% of extracts with some anti-leishmanial activity.	[97]

PTR1: Pteridine reductase 1; APRT: adenine phosphoribosyltransferase; LPG: lipophosphoglycan; GP63: Glycoprotein 63; NA: Not Assessed.

Many compounds tested *in vitro* demonstrated direct activity against *Leishmania* while others targeted the cellular pathways of host cells to control the parasite (Table 7). Some compounds inhibited essential parasite enzymes, such as arginase [55, 82, 83], or trypanothione reductase [51] or both [56]. Other plants extracts exerted their effect through cytokine modulation to enhance immune responses. For instance, *Tetradenia riparia* essential oil increased IFN-g secretion [24]. Modulation of phagocytic activity has also been identified as a mechanism of action including enhancement of NO secretion and phagocytic activity [79, 95] or modification in the activation state of macrophages [18]. ROS production, known to be essential in intracellular parasite clearance, was also commonly observed [18, 19, 62]. While most studies highlighted compounds that act directly against *Leishmania*, several reported anti-leishmanial activity without detailing the underlying mechanisms [7, 11, 97, 104], making it challenging to develop these compounds as potential treatments for *Leishmania*.

## Discussion

This review focuses on identifying specific strategies for drug development by examining recent advances in drug discovery. The WHO currently recommended monotherapies with various approved molecules as first-line treatments, depending on the region. Among these, L-AMB is one of the most effective options; however, it requires hospitalization, is costly, and is difficult to access in many countries. Recent reports of resistant strains highlight the urgent need for innovative drug development strategies to avert a crisis akin to antibiotic resistance.

To address drug resistance, combining authorized drugs has emerged as a strategy. Combinations often reduce dosage, toxicity, and costs, while increasing efficacy. However, some combinations, such as L-AMB with SSG or miltefosine [109] or combination of intralesional Glucantime^®^ with oral itraconazole [8] showed no benefit over monotherapy. Combining miltefosine with pentamidine resulted in additive effects, but also increased side effects and costs, rendering it impractical [93]. Developing effective combinations still requires extensive trials to optimize dosing and efficacy. While lower doses can reduce cytotoxicity and costs, these challenges remain inherent to the molecules themselves and require alternative compounds to resolve fully.

Nanocarriers, such as liposomes, have successfully reduced toxicity in AMB formulations. However, the cost increased due to reliance on animal-derived cholesterol. Using plant-derived lipids like stigmasterol (DSHemsPC) has been proposed as a promising alternative. In mice, this formulation maintained AMB levels in target organs and reduced inflammation and parasite burden [43]. Nanoparticles also enable targeted delivery, delayed release, and modified administration routes [7, 11, 97, 104]. For example, nanoparticles have facilitated oral delivery of combinations such as AMB and paromomycin [73] or topical application of AMB with miltefosine [21]. They are versatile tools for improving treatments, though they significantly increase costs.

Repositioning drugs has proven valuable in neglected tropical diseases. Repositioning saves time and money, as approved drugs have existing safety data. Miltefosine is one of the successful examples. Among repositioned drugs, only fexinidazole has undergone clinical trials for leishmaniasis. Although promising in mice, it led to relapses, causing the trial to be terminated due to insufficient efficacy [74]. However, predicting the effects of immunomodulatory drugs remains challenging. For example, simvastatin reduced parasite burden in both susceptible BALB/c and resistant C57/Bl6 mice, while pravastatin improved outcomes in susceptible mice, but worsened outcomes in resistant ones [72].

Since immunity is critical for disease control, combining immunomodulating compounds with traditional treatments has been proposed. Strategies aim to stimulate pro-inflammatory responses or reduce excessive inflammation, especially in cutaneous lesions. While some approaches have reduced treatment duration and side effects, others have shown no effect [56, 58] or increased adverse reactions as seen with pentoxifylline supplementation [13]. Combining imiquimod with Glucantime^®^ improved cure rates in *L. major* infections [98], but pairing it with SSG for *L. braziliensis* showed no statistical significance [58]. Numerous studies have tried to increase the Th1 response by favoring a pro-inflammatory environment, but there have also been trials that inhibited Th1 responses to limit local inflammation and reduce tissue damage in response to cutaneous infection [53, 85]. Although a promising strategy, immunomodulation requires further development and cannot replace chemotherapy alone.

Ethnobotany uses local knowledge to identify plants with anti-leishmanial potential. Surveys often reveal plants with broad applications, such as treating inflammation or microbial infection [22]. However, not all studies confirm traditional uses. Some extracts exhibit high cytotoxicity, rendering them unsuitable for macrophages [30, 70]. Testing on intracellular amastigotes is critical, as Girardi et al. demonstrated that compounds active against promastigotes and axenic amastigotes failed against intracellular macrophages [34]. Few studies elucidate the mechanism of action, and effects are often attributed to synergistic interactions among multiple compounds. Despite these challenges, ethnobotany remains promising. It supports biodiversity and offers cost-effective options for natural product development. Commercializing these products aligns with growing demand for safer, environmentally conscious treatments.

## Conclusion

In addition to strategies such as combining treatments, using nanoparticle formulations, repositioning drugs, employing immunomodulators, and conducting ethnobotanical research, artificial intelligence (AI) has emerged as a powerful tool in drug discovery. AI can accelerate the identification and optimization of new compounds by predicting molecular properties, optimizing chemical structures, and simulating biological interactions. Finally, AI has the potential to enhance the efficiency of drug development, while significantly reducing the time and cost of research and development.
